# Long noncoding RNA lincRNA-p21 is the major mediator of UVB-induced and p53-dependent apoptosis in keratinocytes

**DOI:** 10.1038/cddis.2015.67

**Published:** 2015-03-19

**Authors:** J R Hall, Z J Messenger, H W Tam, S L Phillips, L Recio, R C Smart

**Affiliations:** 1 Department of Biological Sciences, North Carolina State University, Raleigh, NC, USA; 2 Center for Human Health and the Environment, North Carolina State University, Raleigh, NC, USA; 3 Toxicology Program, North Carolina State University, Raleigh, NC, USA; 4 ILS, Research Triangle Park, NC, USA

## Abstract

LincRNA-p21 is a long noncoding RNA and a transcriptional target of p53 and HIF-1α. LincRNA-p21 regulates gene expression in *cis* and *trans*, mRNA translation, protein stability, the Warburg effect, and p53-dependent apoptosis and cell cycle arrest in doxorubicin-treated mouse embryo fibroblasts. p53 plays a key role in the response of skin keratinocytes to UVB-induced DNA damage by inducing cell cycle arrest and apoptosis. In skin cancer development, UVB-induced mutation of p53 allows keratinocytes upon successive UVB exposures to evade apoptosis and cell cycle arrest. We hypothesized that lincRNA-p21 has a key functional role in UVB-induced apoptosis and/or cell cycle arrest in keratinocytes and loss of lincRNA-p21 function results in the evasion of apoptosis and/or cell cycle arrest. We observed that lincRNA-p21 transcripts are highly inducible by UVB in mouse and human keratinocytes in culture and in mouse skin *in vivo*. LincRNA-p21 is regulated at the transcriptional level in response to UVB, and the UVB induction of lincRNA-p21 in keratinocytes and *in vivo* in mouse epidermis is primarily through a p53-dependent pathway. Knockdown of lincRNA-p21 blocked UVB-induced apoptosis in mouse and human keratinocytes, and lincRNA-p21 was responsible for the majority of UVB-induced and p53-mediated apoptosis in keratinocytes. Knockdown of lincRNA-p21 had no effect on cell proliferation in untreated or UVB-treated keratinocytes. An early event in skin cancer is the mutation of a single p53 allele. We observed that a mutant p53^+/R172H^ allele expressed in mouse epidermis (K5Cre^+/tg^;LSLp53^+/R172H^) showed a significant dominant-negative inhibitory effect on UVB-induced lincRNA-p21 transcription and apoptosis in epidermis. We conclude lincRNA-p21 is highly inducible by UVB and has a key role in triggering UVB-induced apoptotic death. We propose that the mutation of a single p53 allele provides a pro-oncogenic function early in skin cancer development through a dominant inhibitory effect on UVB-induced lincRNA-p21 expression and the subsequent evasion of UVB-induced apoptosis.

The Human Genome Project revealed that only ~3% of human genome encodes protein.^1^ The remaining 97% of the human genome is referred to as noncoding DNA. Initially, much of the intergenic noncoding sequence was referred to as ‘junk DNA' as it was considered to have no function. Although some intergenic sequences contain DNA elements important in gene regulation, it is now known that many intergenic sequences can be transcribed into RNA.^2, 3, 4, 5^ In fact, ~85% of the human genome is transcribed into RNA.^6, 7^ RNA transcripts that lack protein-coding function are referred to as noncoding RNAs (ncRNAs) and of these the long ncRNAs (lncRNAs; ≥200 nt) represent the majority. Most lncRNAs are transcribed from intergenic or intronic regions of the genome or overlap with or are transcribed antisense to protein-coding genes.^8^ LncRNAs are one of the largest and more diverse classes of cellular transcripts with over 10 000 lncRNA transcripts reported in the human genome.^6, 7^

Only a handful of lncRNAs have been studied to date and mostly in cell culture. These lncRNAs are involved in regulating gene expression through a variety of mechanisms including epigenetic silencing, transcriptional regulation, RNA processing, RNA modification and translation.^4, 9^ Emerging evidence indicates that lncRNAs are associated with human diseases such as cancer,^10, 11^ Alzheimer's^12^ and heart disease.^13^ In lung, liver, prostate and breast cancer, the expression of certain lncRNAs correlates with tumor development, progression or survival.^10, 11^ Half of all the trait-associated SNPs identified in GWAS are located in non-coding DNA intergenic sequences, and many of the intergenic regions may function by encoding lncRNAs.^14^ These results point to important roles of lncRNAs in human disease. It is critical to determine whether associations of lncRNAs with specific diseases are functionally significant and to develop mouse genetic models to define and characterize the function of lncRNAs in disease *in vivo*. LncRNAs could represent diagnostic markers and/or possible therapeutic targets. Moreover, because the etiology of most chronic human diseases involves interactions with the environment,^15^ it is important to determine whether environmental factors can impact the expression, activity and function of lncRNAs to contribute to disease pathogenesis.

Nonmelanoma skin cancer (NMSC) is the most common cancer in the United States.^16^ The majority of NMSCs is environmentally induced and caused by solar UVB radiation which produces DNA damage and mutations. Each year, there are more cases of NMSC than all cases of breast, prostate, lung and colon cancers combined. For skin squamous cell carcinomas (SCCs), the incidence of p53 mutations ranges from 50 to 90% in both humans and mice.^17, 18^ UVB-induced mutation of p53 allows keratinocytes upon successive UVB exposures to evade apoptosis^19, 20, 21, 22^ and cell cycle arrest.^23^ These defects have a critical role in skin cancer development. Mice lacking one or both copies of p53 as well as mutant p53 mice all display increased susceptibility to UVB-induced skin cancer^20, 24, 25, 26, 27^ and greatly decreased apoptosis in response to UVB.^19, 20, 21, 22^

LincRNA-p21 is a long intergenic non-coding RNA (3100 nt).^28^ Because of lincRNA-p21's location on chromosome 17, approximately 15 kb upstream from the *Cdkn1a* (p21) gene, it was named lincRNA-p21.^28^ LincRNA-p21 was first reported to be a direct transcriptional target of p53 and to mediate p53-dependent apoptosis but not cell cycle arrest in doxorubicin-treated mouse embryo fibroblasts (MEFs).^28^ Another more recent report indicates that lincRNA-p21 has no role in apoptosis and has an important role in regulating p53-dependent cell cycle arrest in doxorubicin-treated MEFs.^29^ The former report^28^ states that lincRNA-p21 regulates global gene expression in trans whereas the latter report^29^ indicates that lincRNA-p21 only regulates nearby *Cdkn1a* (p21) in a *cis* manner. Thus, there are conflicting reports on the role of lincRNA-p21 in MEFs. LincRNA-p21 can also regulate mRNA translation and protein stability.^30^ Recently, LincRNA-p21 transcripts were shown to be upregulated in livers of mice treated with the carcinogen furan^31^ and lincRNA-p21 was shown to be hypoxia-responsive and promote glycolysis and regulate the Warburg effect independent of p53.^32^

Because of the key role of p53 in UVB-induced apoptosis, cell cycle arrest and skin cancer, we reasoned that lincRNA-p21 could have a critical functional role in UVB-induced apoptosis and/or cell cycle arrest in keratinocytes and its loss in the evasion of apoptosis and/or defective cell cycle control and the pathogenesis of skin cancer. Our results reveal that lincRNA-p21 is highly inducible by UVB through a p53-dependent mechanism and that lincRNA-p21 has a key role in triggering UVB-induced apoptosis in human and mouse keratinocytes.

## Results

### LincRNA-p21 transcripts are highly inducible by UVB in mouse and human keratinocytes in culture and in mouse skin *in vivo*

**Figure 1:**
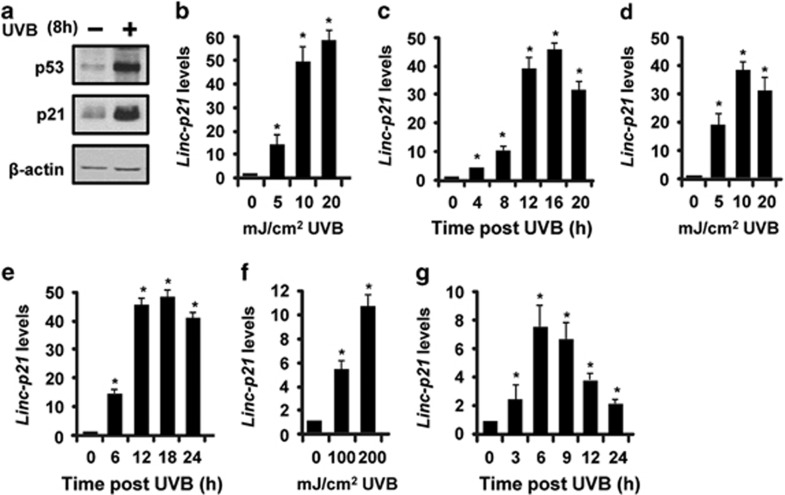
LincRNA-p21 (Linc-p21) transcripts are highly inducible by UVB in mouse and human keratinocytes in culture and in mouse skin. (**a**) Balb/MK2 mouse keratinocytes were exposed to UVB, collected 8 h later and immunoblot analysis conducted. (**b**) Balb/MK2 mouse keratinocytes were exposed to the indicated doses of UVB, collected 16 h later and lincRNA-p21 transcript levels measured. (**c**) Balb/MK2 cells were exposed to 10 mJ/cm^2^ UVB and lincRNA-p21 transcript levels measured at the indicated times. (**d**) NHEK cells were exposed to the indicated doses of UVB, collected 16 h later and lincRNA-p21 transcripts measured. (**e**) NHEK cells were exposed to 10 mJ/cm^2^ UVB and lincRNA-p21 transcripts measured at the indicated times. (**f**) SKH-1 mice (three mice per time point) were exposed to 100 or 200 mJ/cm^2^ UVB, epidermis was collected at 9 h and lincRNA-p21 levels measured. (**g**) SKH-1 mice (three mice per group) were treated with 100 mJ/cm^2^ and lincRNA-p21 transcripts measured at the indicated times. LincRNA-p21 levels were measured by TaqMan real-time PCR (Ct value at peak post UVB=26–29 cycles using 25 ng template for all experiments). LincRNA-p21 was normalized to *β*-actin. Data are expressed as the mean ±S.D. *N*≥3, *P <0.05 significantly different compared with time 0 using student *t*-test

Treatment of Balb/MK2 mouse keratinocytes with UVB radiation resulted in increased levels of p53 protein and its target gene, p21 (Figure 1a) demonstrating that Balb/MK2 keratinocytes are responsive to UVB radiation. To determine whether UVB radiation can increase lincRNA-p21 transcript levels, Balb/MK2 keratinocytes were treated with various doses of UVB radiation. UVB exposure was an potent inducer of lincRNA-p21 transcripts producing up to a ~60-fold increase in lincRNA-p21 (Figure 1b). UVB treatment increased lincRNA-p21 in a dose-dependent manner (Figure 1b). Time-course studies revealed that UVB increased lincRNA-p21 levels as early as 4 h post UVB treatment with peak transcript levels occurring at ~16 h (Figure 1c). Normal human epidermal keratinocytes (NHEK) also displayed a UVB dose-dependent induction of lincRNA-p21 (Figure 1d) and a similar time course and magnitude of induction of lincRNA-p21 (~50-fold) (Figure 1e) as mouse keratinocytes. To determine whether UVB radiation was capable of inducing lincRNA-p21 *in vivo* in skin (epidermis), we utilized SKH-1 hairless mice and environmentally relevant doses of UVB. SKH-1 mice are an experimental model used to study the effects of UVB in skin and are relevant to UVB-induced human SCC skin cancer as the UV-induced tumors in these mice resemble, both at the morphologic and molecular levels, the UVB-induced SCC skin cancers in humans.^33^ The minimal erythema dose (MED) of UVB treatment in skin is defined as the minimal dose that produces a just-perceptible erythema (redness) at 24 h. SKH-1 mice were treated with UVB doses that correspond to 0.5 (100 mJ/cm^2^) and 1.0 MED (200 mJ/cm^2^). LincRNA-p21 was highly inducible by UVB radiation in SKH-1 mouse epidermis *in vivo* (Figures 1f and g). Collectively, these data demonstrate that lincRNA-p21 transcripts are highly inducible by UVB in mouse and human keratinocytes in culture and in mouse skin *in vivo*.

### LincRNA-p21 is transcriptionally upregulated in response to UVB and is p53-dependent in keratinocytes in culture and in mouse skin *in vivo*

**Figure 2:**
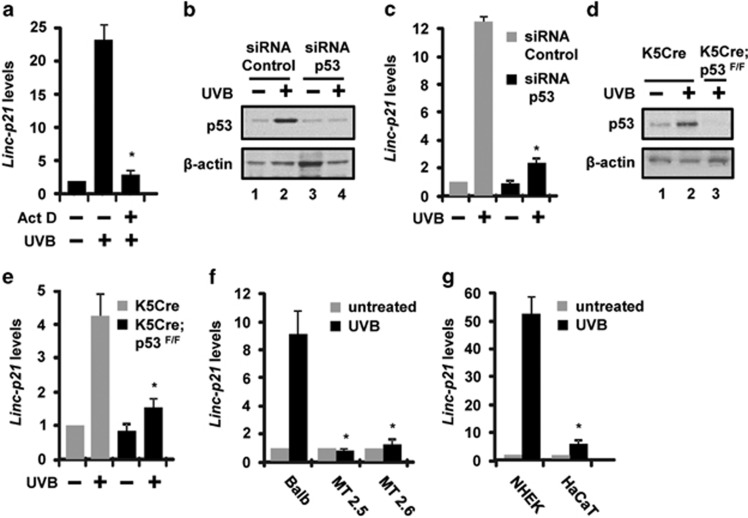
LincRNA-p21 is regulated at the transcriptional level and is p53-dependent in mouse and human keratinocytes and mouse skin *in vivo*. (**a**) Balb/MK2 keratinocytes were treated with actinomycin D, exposed to 10 mJ/cm^2^ UVB, collected 12 h later and lincRNA-p21 transcripts measured. (**b**) Balb/MK2 cells were transfected with siRNA to p53 or control siRNA, and 48 h post transfection, cells were exposed to 10 mJ/cm^2^ UVB, collected 12 h later and p53 protein levels were measured by immunoblot analysis. (**c**) Balb/MK2 cells were transfected with siRNA to p53 or control siRNA, and 48 h post transfection, cells were exposed to 10 mJ/cm^2^ UVB, collected 12 h later and lincRNA-p21 transcripts measured. (**d**) K5Cre^+/tg^ and K5Cre^+/tg^;p53^flox/flox^ SKH-1 mice were treated with 200 mJ/cm^2^ UVB and epidermis collected 9 h later and p53 protein levels were measured by immunoblot analysis. (**e**) K5Cre^+/tg^ and K5Cre^+/tg^;p53^flox/flox^ mice were treated with 200 mJ/cm^2^ UVB and epidermis collected 9 h later and lincRNA-p21 transcripts measured. (**f**) MT2.5, MT2.6 and Balb/MK2 cells were exposed to 10 mJ/cm^2^ UVB and collected 8 h later. (**g**) NHEK and HaCaT cells were exposed to 10 mJ/cm^2^ UVB and collected 12 h later. LincRNA-p21 transcript levels were determined by TaqMan real-time PCR. Expression of lincRNA-p21 was normalized to *β*-actin. Data expressed in **a**, **c**, **e**, **f** and **g** as the mean ±S.D. *N* ≥3, *P<0.05 significantly different compared to UVB exposed control as determined by the student *t*-test

To determine whether lincRNA-p21 is regulated at the transcriptional level in response to UVB treatment, we treated Balb/MK2 keratinocytes with actinomycin D, an inhibitor of transcription, and then exposed the cells to UVB and collected the cells 12 h later. As shown in Figure 2a, actinomycin D blocked the increase in UVB-induced lincRNA-p21 transcripts indicating that lincRNA-p21 is regulated at the transcriptional level in response to UVB treatment. Next, we examined the role of p53 on the regulation of UVB-induced lincRNA-p21 expression in Balb/MK2 keratinocytes in culture. UVB-treated p53 knockdown keratinocytes (Figure 2b) were significantly impaired in their ability to induce lincRNA-p21 transcripts (Figure 2c). Next, we examined the role of p53 on the regulation of UVB-induced lincRNA-p21 expression *in vivo* in mouse epidermis using K5Cre^+/tg^;p53^flox/flox^ mice. In this mouse model, the keratin 5 (K5) promoter directs Cre recombinase expression to the epidermis.^34^ UVB-treated K5Cre^+/tg^;p53^flox/flox^ mice SKH-1 mice lacking p53 in their epidermis (Figure 2d) displayed significantly decreased levels of epidermal lincRNA-p21 transcripts compared with UVB-treated K5Cre^+/tg^ mice (Figure 2e). Although the majority (~85%) of UVB-induced lincRNA-p21 in mouse skin *in vivo* occurs through a p53-dependent pathway, there also appears to be a minor (~15%) p53-independent pathway involved in the UVB regulation of lincRNA-p21 transcript levels (Figures 2e). We also observed that mouse skin SCC lines that are defective in p53 signaling (MT2.5 and MT2.6)^35^ were unable to induce lincRNA-p21 in response to UVB radiation compared with mouse keratinocytes (Figure 2f). Likewise, HaCaT cells which are a spontaneously immortalized human keratinocyte cell line that contain two alleles of mutant p53^36^ also displayed significantly impaired UVB induction of lincRNA-p21 when compared with similarly treated NHEKs (Figure 2g). Collectively, these data demonstrate that lincRNA-p21 is regulated at the transcriptional level in response to UVB treatment and that the UVB induction of lincRNA-p21 in keratinocytes and *in vivo* in mouse epidermis is dependent upon p53.

### LincRNA-p21 is a critical regulator of apoptosis in UVB-treated mouse and human keratinocytes

**Figure 3:**
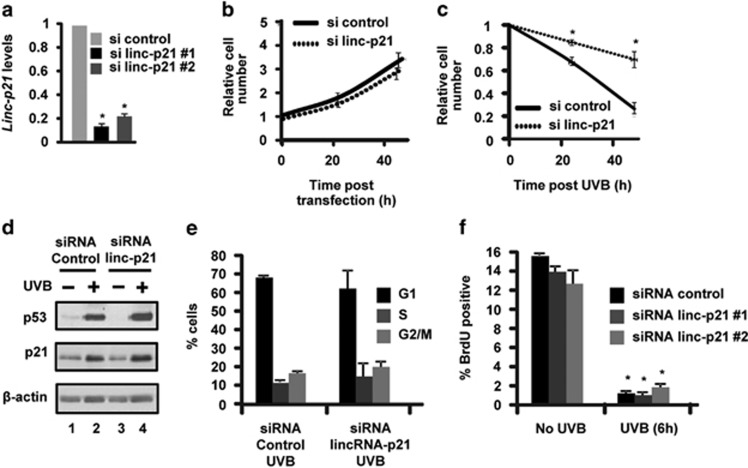
LincRNA-p21 (linc-p21) does not regulate cell proliferation in UVB-treated keratinocytes. (**a**) Balb/MK2 cells were transfected with siRNA to lincRNA-p21 or control siRNA, and 48 h post transfection, cells were exposed to 10 mJ/cm^2^ UVB, collected 18 h later and lincRNA-p21 transcripts measured. (**b**) Control siRNA and lincRNA-p21 #1 knockdown Balb/MK2 cells were counted at 0, 24 and 48 h post transfection. (**c**) Control and lincRNA-p21 #1 knockdown Balb/MK2 cells were exposed to 10 mJ/cm^2^ UVB (48 h post transfection, *t*=0), and cells were counted 24 and 48 h after UVB (*N*=3 for each time point). (**d**) Control and lincRNA-p21 #1 knockdown Balb/MK2 cells were exposed to 10 mJ/cm^2^ UVB (48 h post transfection) and collected 18 h after UVB and immunoblot analysis conducted. (**e**) Control and lincRNA-p21 #1 knockdown Balb/MK2 cells were exposed to 10 mJ/cm^2^ UVB (48 h post transfection) and collected 24 h after UVB, PI-stained and analyzed by FACS. (**f**) Control and lincRNA-p21 #1 and #2 knockdown Balb/MK2 cells were exposed to 10 mJ/cm^2^ UVB (48 h post transfection) and pulse-labeled with BrdU for 1 h and collected 6 h after UVB. Cells were stained with BrdU-FITC and PI-stained and analyzed by FACS. Data expressed in **a**, **b**, **c**, **e** and **f** as the mean ±S.D. *N* ≥3, *P<0.05 significantly different compared with UVB-exposed control as determined by the student *t*-test

**Figure 4:**
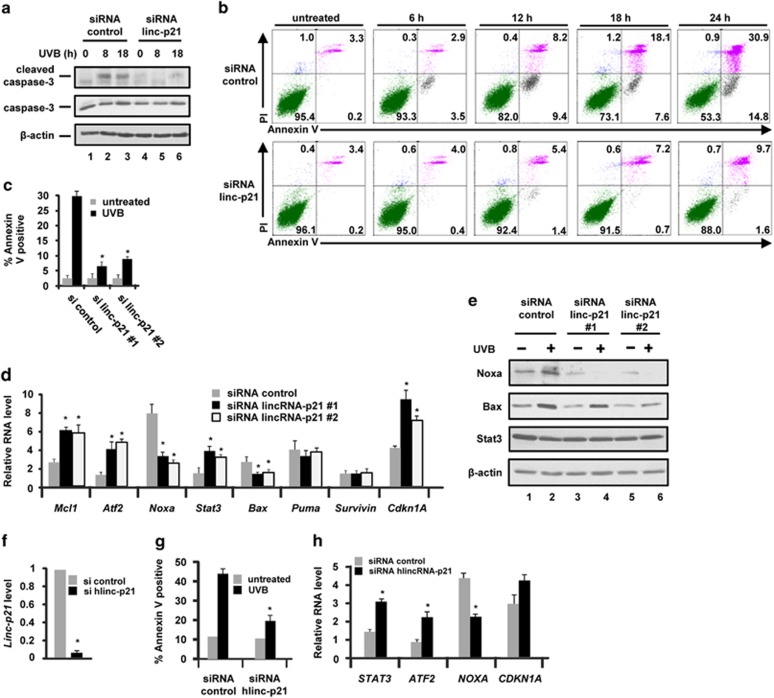
LincRNA-p21 (linc-p21) regulates apoptosis in UVB-treated keratinocytes. (**a**) Control and lincRNA-p21 #1 knockdown Balb/MK2 cells were exposed to 10 mJ/cm^2^ UVB (48 h post transfection) and collected at 0, 8 and 18 h after UVB exposure and immunoblot analysis for caspase 3 conducted; results shown are representative of duplicate experiments. (**b**) Control and lincRNA-p21 #1 knockdown Balb/MK2 cells were exposed to 10 mJ/cm^2^ UVB (48 h post transfection) and collected at 0, 6, 12, 18 and 24 h after UVB. Cells were stained with annexin-V and PI and analyzed by FACS. Similar results were obtained in three independent experiments. (**c**) Control and lincRNA-p21 #1 and #2 knockdown Balb/MK2 cells were exposed to 10 mJ/cm^2^ UVB (48 h post transfection) and 24 h after UVB. Cells were stained with annexin-V and PI and analyzed by FACS. Similar results were obtained in three independent experiments. (**d**) Control and lincRNA-p21 #1 and #2 knockdown Balb/MK2 cells were exposed to 10 mJ/cm^2^ and collected at 18 h post UVB and candidate gene expression examined by Taqman RT-PCR. (**e**) Control and lincRNA-p21 #1 and #2 knockdown Balb/MK2 cells were exposed to 10 mJ/cm^2^ and collected at 18 h post UVB and immunoblot analysis conducted. (**f**) NHEK cells were transfected with siRNA to human lincRNA-p21 or control siRNA, 48 h later exposed to 10 mJ/cm^2^ UVB, collected 18 h later and lincRNA-p21 levels measured. (**g**) Control and lincRNA-p21 knockdown NHEK cells were exposed to 10 mJ/cm^2^ UVB (48 h post transfection) and collected at 0 and 18 h after UVB. Cells were stained with annexin-V and PI and analyzed by FACS. Similar results were obtained in three independent experiments. (**h**) Control and lincRNA-p21 knockdown NHEK cells were exposed to 10 mJ/cm^2^ and collected at 18 h post UVB, and candidate gene expression examined. Data are expressed in **c**, **d**, **f**, **g** and **h** as the mean ±S.D. *N* ≥3, *P<0.05 significantly different compared with siRNA control as determined by the student *t*-test

To begin to determine the functional role of lincRNA-p21 in UVB-treated keratinocytes, we used an siRNA approach to knockdown lincRNA-p21. As shown in Figure 3a, we were able to knockdown lincRNA-p21 transcript levels in UVB-treated mouse keratinocytes by >80% using two different siRNA sequences.^26^ Knockdown of lincRNA-p21 had no effect on cell viability in untreated keratinocytes (Figure 3b); however, UVB-treated lincRNA-p21-depleted keratinocytes displayed an increased viability (Figure 3c). These results suggest that in UVB-treated keratinocytes, lincRNA-p21 functions to inhibit keratinocyte cell cycle progression and/or to induce keratinocyte apoptosis in response to UVB. Earlier studies showed that lincRNA-p21 has an important role in regulating p53-dependent cell cycle arrest involving p21 in doxorubicin-treated MEFs.^29^ Therefore, we first examined the levels of p53 and p21 in control and lincRNA-p21-depleted keratinocytes and observed that the levels of p53 and p21 were not affected by the knockdown of lincRNA-p21 (Figure 3d). These results indicate that the knockdown of lincRNA-p21 does not interfere with p53 protein levels or the regulation of p21 in response to UVB. In accord with these results, the knockdown of lincRNA-p21 had no effect on the cell cycle distribution and the DNA damage checkpoint as determined by FACS analysis (Figures 3e and f). In contrast to the lack of effect on cell proliferation and DNA damage checkpoint function, lincRNA-p21-depleted keratinocytes displayed striking decreases in UVB-induced apoptosis as determined by decreased cleaved caspase-3 levels (Figure 4a) and decreased annexin-V staining (Figure 4b). On the basis of annexin V single positive staining, there was a ~10-fold decrease in early apoptotic cells in lincRNA-p21-depleted keratinocytes at 18 h post UVB (Figure 4b). Similar results were obtained with a second lincRNA-p21 siRNA sequence (Figure 4c). These data demonstrate that lincRNA-p21 is a critical regulator of UVB-induced apoptosis and regulates at least 75% of the apoptosis induced by UVB in mouse keratinocytes (Figures 4b and c). Knockdown of lincRNA-p21 resulted in altered expression of several genes associated with apoptosis in response to UVB radiation indicating that lincRNA-p21 can both repress the expression of anti-apoptotic and activate the expression of pro-apoptotic genes in response to UVB (Figure 4d). The levels of *Cdkn1a* mRNA were not decreased by knockdown of lincRNA-p21 in UVB-treated keratinocytes; in fact, there was an unexpected ~two fold increase. Additionally, the protein levels of the pro-apopotic factors Noxa and Bax were significantly reduced in lincRNA-p21 knockdown cells following UVB exposure and despite changes in Stat3 mRNA, no changes in Stat3 protein were observed (Figure 4e). Next, we examined the role of lincRNA-p21 in UVB-induced apoptosis in human keratinocytes. The knockdown of human lincRNA-p21 in NHEKs (Figure 4f) resulted in decreased apoptosis as determined by annexin V staining (Figure 4g). Thus, lincRNA-p21 is a key mediator of UVB-induced apoptosis in human keratinocytes and is responsible for ~70% of UVB-induced apoptosis (Figure 4g). Knockdown of lincRNA-p21 in NHEKs resulted in the altered expression of apoptosis-related genes in response to UVB; lincRNA-p21 represses the expression of anti-apoptotic genes and activates the expression of pro-apoptotic genes in response to UVB and had no effect on *CDKN1A* mRNA levels (Figure 4h). These data reveal that lincRNA-p21 has a major and critical role in UVB-induced apoptotic death in human and mouse keratinocytes.

### Mutant p53^+/R172H^ allele displays a significant dominant-negative inhibitory effect on UVB-induced lincRNA-p21 transcription and apoptosis in epidermis

**Figure 5:**
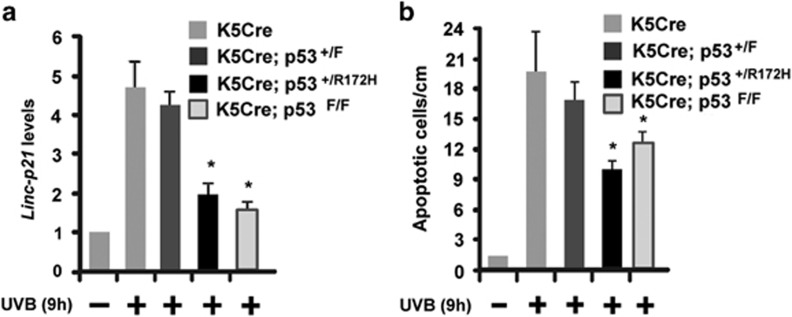
Mutation of single p53 allele has a dominant-negative effect *in vivo* in mouse epidermis. (**a**) K5Cre^+/tg^, K5Cre^+/tg^;LSLp53^+/R172H^, K5Cre^+/tg^;p53^+/flox^ and K5Cre^+/tg^;p53^flox/flox^ SKH-1 mice were treated with 200 mJ/cm^2^ UVB and epidermal lincRNA-p21 transcripts measured 9 h post UVB treatment. (**b**) K5Cre^+/tg^, K5Cre^+/tg^;LSLp53^+/R172H^, K5Cre^+/tg^;p53^+/flox^ and K5Cre^+/tg^;p53^flox/flox^ SKH-1 mice were treated with 100 mJ/cm^2^ UVB and the number of apoptotic interfollicular basal epidermal keratinocytes/cm skin were scored at 9 h post UVB treatment. Data are expressed as the mean ±S.D. *N*≥3, *P <0.05 significantly different compared with UVB-treated K5Cre mice as determined by the student *t*-test

Because the development of UVB-induced skin cancer often entails early oncogenic events involving the mutation of a single p53 allele and the escape from UVB-induced apoptosis, we examined whether a dominant inhibitory mutation in a single p53 allele (p53^+/R172H^) could greatly impair UVB-induced lincRNA-p21 transcript levels and apoptosis when compared with loss-of-function mutation involving deletion of a single p53 allele (p53^+/−^). Previous studies have shown that missense mutants of p53 in the heterozygous state can function as dominant-negative inhibitors of certain tumor-suppressive functions and retain normal wild-type p53 of others.^37, 38^ For example, in p53^+/R172H^ cells, the mutant p53 does not have a dominant-negative effect on DNA damage-induced G_1_ arrest or p21 gene expression but does have a dominant-negative effect on gamma radiation-induced apoptosis.^38^ Therefore, we generated two genetically engineered mouse models (K5Cre^+/tg^;p53^+/flox^ and K5Cre^+/tg^;LSLp53^+/R172H^)^38, 39^ to test whether the mutation of a single p53 allele (p53^+/R172H^) as opposed to the loss of a single p53 allele (p53^+/−^), both expressed under normal physiological control *in vivo* in epidermis of SKH-1 mice, will have a dominant-negative effect on UVB-induced lincRNA-p21 expression and keratinocyte apoptosis. In this mouse model, the keratin 5 (K5) promoter directs Cre recombinase expression to the epidermis.^34^ As shown in Figure 5a, UVB-induced lincRNA-p21 levels in K5Cre^+/tg^;LSLp53^+/R172H^ mice were greatly decreased and were similar to the levels in K5Cre^+/tg^;p53^flox/flox^ mice whereas lincRNA-p21 transcript levels in K5Cre^+/tg^ and K5Cre^+/tg^;p53^+/flox^ were similar. These results demonstrate a potent effect of the dominant inhibitory mutation of a p53 allele (p53^+/R172H^) on UVB-induced lincRNA-p21 transcript levels. Next, we examined whether the decreased levels of lincRNA-p21 transcripts in UVB-treated K5Cre^+/tg^;LSLp53^+/R172H^ are associated with decreased p53-dependent apoptosis induced by UVB. As shown in Figure 5b, approximately 50% of UVB-induced apoptosis in mouse epidermis is dependent on p53. UVB-induced apoptosis was similarly decreased in K5Cre^+/tg^;p53^flox/flox^ and K5Cre^+/tg^;LSLp53^+/R172H^ mice indicating that the mutation in a single p53 allele (p53^+/R172H^) has a significant dominant-negative inhibitory effect on lincRNA-p21 transcription and apoptosis *in vivo*. These data further suggest that deregulation of lincRNA-p21 expression may be important in the early pro-oncogenic functions of p53 mutant keratinocytes during the development of skin cancer.

## Discussion

In sun-exposed areas of the human skin, p53 plays an important role in skin cancer prevention through its regulation of keratinocyte cell cycle arrest and apoptosis in response to UVB radiation-induced DNA damage.^17, 19, 20, 21, 22, 23, 24, 40^ These p53-regulated responses serve to prevent UVB-induced mutagenesis by producing cell cycle arrest to allow time for DNA repair and by triggering apoptotic death of DNA-damaged keratinocytes. We have identified lincRNA-p21 as the key mediator of UVB-induced apoptosis in human and mouse keratinocytes. Our results demonstrate that p53 plays a key role in regulating lincRNA-p21 transcript levels in response to UVB in keratinocytes and that lincRNA-p21 has a critical function in triggering apoptosis in UVB-treated keratinocytes. In fact, lincRNA-p21 is responsible for the majority of UVB-induced apoptosis in both human and mouse keratinocytes.

Several lncRNAs have been shown to be regulated by p53 or to modulate apoptosis in response to DNA damage, for example, Pint^41^ and Panda^42^ have anti-apoptotic activity, whereas INXS,^43^ MEG3,^44^ lincRNA-p21^28^ and Gas5^45^ all have pro-apoptotic activity. It is generally considered that p53-regulated lncRNAs serve to fine tune p53-regulated apoptosis and/or cell cycle arrest and function as low copy number lncRNAs. Our results indicate that lincRNA-p21 does much more than fine tune the p53 response in UVB-treated keratinocytes as lincRNA-p21 is responsible for the majority of UVB-induced and p53-mediated apoptosis. LincRNA-p21 is regulated at the transcriptional level in response to UVB and the UVB induction of lincRNA-p21 in keratinocytes and *in vivo* in mouse epidermis is primarily through a p53-dependent pathway. We observed that UVB is an extremely potent inducer of lincRNA-p21, producing ~50–60-fold increase in mouse and human keratinocytes. Thus, lincRNA-p21 transcript levels rise from low copy number of transcripts to very significant transcript levels in response to UVB to trigger apoptotic keratinocyte death. Evaluation of candidate gene expression in UVB-treated keratinocytes revealed that lincRNA-p21 represses the expression of anti-apoptotic genes Mcl1, Stat3 and Atf2 and activates the expression of pro-apoptotic genes Noxa and Bax. Future studies are required to understand how lincRNA-p21 is repressing and activating gene expression in keratinocytes in response to UVB treatment.

As mentioned in the 'Introduction', there are conflicting reports on the functional role for lincRNA-p21 in MEFS. Huarte *et al.*^28^ reported that lincRNA-21 functions as the global *trans* regulator of gene expression, has no effect on p21 expression and mediates p53-dependent apoptosis but not cell cycle arrest in doxorubicin-treated MEFs. On the other hand, Dimitrova *et al.*^29^ report lincRNA-p21 does not regulate apoptosis but regulates p53-mediated cell cycle arrest through the regulation of p21 in *cis* in doxorubicin-treated MEFs and that lincRNA-p21 indirectly regulates genes associated the Polycomb Repressive Complex 2 through a p21-dependent mechanism. A major technical difference between these studies is that Huarte *et al.*^28^ used an RNAi knockdown approach and Dimitorva *et al.^29^*employed knockout MEFs. Dimitorva *et al.* suggested that this difference in depleting lincRNA-p21 could be responsible for the different cellular outcomes. Our results agree with the findings of Huarte *et al.*^28^ and like their study, our studies utilized an RNAi knockdown approach. We found lincRNA-p21 has a key role in regulating UVB- and p53-mediated apoptosis in both human and mouse keratinocytes using three different RNAis. These results argue against off-target effects and further studies in lincRNA-p21 knockout keratinocytes will be required to address method of depletions and whether lincRNA-p21 functions in *cis* or *trans* to regulate gene expression in UVB-treated keratinocytes.

Depletion of lincRNA-p21 allows keratinocytes to evade UVB-induced apoptosis suggesting a possible tumor suppressor function for lincRNA-p21 in NMSC. In skin cancer, the incidence of p53 mutations ranges from 50 to 90% in humans and mice.^17, 18^ Mutant p53 provides keratinocytes an advantage over normal keratinocytes in response to successive UVB exposure by evading cell cycle checkpoints and by allowing mutant keratinocytes to evade UVB-induced apoptosis.^17, 19, 20, 21, 22, 23, 40^ These events promote genomic instability, clonal expansion and the development of skin cancer. In early skin cancer development, a single p53 allele is mutated or deleted as an early initiating oncogenic event.^17^ Patches/clones of mutant p53 keratinocytes (considered to be the precursor lesion to skin cancer) can be detected in sun-exposed areas of human skin and in UVB-treated mouse skin long before tumors form.^17, 46, 47^ Most of these clones have a single mutant allele of p53 (97% missense and 3% nonsense).^17^ Collectively, these studies indicate that mutation of a single p53 allele provides a pro-oncogenic function/advantage early in skin cancer development. Our results demonstrate a potent dominant-negative inhibitory effect of a single mutant p53 allele (p53^+/R172H^) on UVB-induced lincRNA-p21 transcript levels and UVB-induced apoptosis. We propose that the mutation of a single p53 allele provides a pro-oncogenic function early in skin cancer development through a dominant inhibitory effect on UVB-induced lincRNA-p21 expression and the subsequent evasion of UVB-induced apoptosis.

The etiology of most chronic human diseases involves complex interactions among environmental factors and an individual's genetic and epigenetic makeup. However, these gene × environment interactions are poorly understood, leading to a deficit in our understanding of how these interactions contribute to adverse health outcomes. Our study demonstrates for the first time that exposure to an environmental factor, in this case solar UVB radiation, can impact the expression, activity and function of a lncRNA. Moreover, LincRNA-p21 expression is induced *in vivo* in mouse skin by environmentally relevant doses of UVB. We speculate that lincRNA-p21 may function as a tumor suppressor gene in UVB-induced non-melanoma skin cancer where the loss of lincRNA-p21 expression results in the evasion of apoptosis.

## Materials and Methods

### Cells and mice

Balb/MK2 keratinocytes were a gift from B. E. Weissman (UNC)^48^ are maintained in Ca^2+^-free EMEM (Lonza, Walkersville, MD, USA), 8% Chelax-treated FBS (F2442, Sigma Aldrich, St. Louis, MO, USA), 4 ng/ml hEGF (Life Technologies, Carlsbad, CA, USA) and 0.05 mM CaCl_2._^48^ NHEKs were purchased from Lonza and maintained in KGM-Gold keratinocyte medium (Lonza). 129S-p53LSL.R172H (LSLp53^+/R172H^) mice were purchased from The Jackson Laboratory (Bar Harbor, ME, USA).^38^ FVB.129-Trp53^tm1Brn^ (p53^flox/flox^) were obtained through the NCI Mouse Repository.^39^ To generate SKH-1 mice, LSLp53^+/R172H^ and p53^flox/flox^ male mice were mated to SKH-1 (Charles River Labs, Wilmington, MA, USA) females. Male LSLp53^+/R172H^ and p53^flox/flox^ males were backcrossed to SKH-1 females five times to obtain SKH-1 LSLp53^+/R172H^ and p53^flox/flox^ SKH-1 mice. K5Cre, K5Cre;LSLp53^+/R172H^, K5Cre;p53^+/Flox^ and K5Cre;p53^flox/flox^ littermates that were used in this study were obtained by crossing LSLp53^+/R172H^ and p53^+/flox^ SKH-1 female mice with K5Cre;LSLp53^+/R172H^ and K5Cre;p53^+/flox^ SKH-1 male mice. K5Cre mice were a gift from Angel Ramirez and Jose Jorcano^34^ and detailed information on control (K5Cre^+/tg^) SKH-1 mice have been described.^50^ All aspects of animal care and experimentation described in this study were conducted according to the NIH guidelines and approved by the Institutional Animal Care and Use Committee of NCSU.

### UVB treatment and chemicals

Balb/MK2 keratinocytes were exposed at less than 50% confluence to 10 mJ/cm^2^ UVB with a calibrated UVB lamp as previously described.^49^ SKH-1 mice were treated with a single dose of 100 or 200 mJ/cm^2^ UVB as described.^50^

### Small interfering RNA

siRNA targeting mouse p53 (5′-AGAAGAAAAUUUCCGCAAAdTdT-3′), mouse LincRNA-p21 #1 (5′- UGAAAAGAGCCGUGAGCUAdTdT-3′),^26^ mouse LincRNA-p21 #2 (5′- AAATAAAGATGGTGGAATGdTdT-3′), human LincRNA-p21 (5′- CUGCAAGGCCGCAUGAUGAdTdT-3′) and the negative control (GFP, 5′-GGCUACGUCCAGGAGCGCACCdTdT-3′) were synthesized by Life Technologies and transfected at the final concentration of 100 nM. All transfections were performed using DharmaFECT Reagent 1 (GE Healthcare, Little Chalfont, UK) according to the manufacturer's recommendations. Cells were exposed to UVB 48 h post siRNA transfection.

### Preparation of epidermal lysates and nuclear cell extracts

Mice were killed by cervical dislocation and dorsal skin was removed and subjected to 6 s heat shock in 60 °C dH_2_0 followed by 10 s in ice water. Epidermis was isolated and RNA was purified as described below. Protein lysates were prepared in RIPA buffer (1% NP-40, 0.5% sodium deoxycholate, 0.1% sodium dodecyl sulfate, 1 mM dithiothreitol, 1 mM sodium orthovanadate, 1 mM AEBSF, and 1 × protease inhibitor cocktail (Roche, Indianapolis, IN, USA) in PBS. Lysates were sonicated and centrifuged at 14 000 × g for 10 min. Equal protein was resolved by SDS-PAGE.

### Antibodies

Antibodies against p21 (sc-471) and Bax (sc-493) were purchased from Santa Cruz Biotechnology (Santa Cruz, CA, USA). Noxa antibody (ab13654) was purchased from Abcam (Cambridge, UK). p53 antibody (1C12) was purchased from Cell Signaling (Danvers, MA, USA). *β*-Actin antibody (A5441) was purchased from Sigma Aldrich. Caspase-3 antibody (CS-9665S) and cleaved caspase-3 (Cleaved Caspase-3 (CS-9661S)) were purchased from Cell Signaling and generously provided by Dr. Jun Tsuji (NCSU).

### RNA and quantitative PCR

Total RNA was isolated using QiaZOL lysis (Qiagen, Valencia, CA, USA), and further purified using Qiagen RNeasy columns. cDNA was prepared from RNA by ImProm-II Reverse Transcription System (Promega, Madison, WI, USA). Quantitative PCR for mouse LincRNA-p21 (FAM probe TGGCCAAACACTGGTG, forward primer GAAGCTTCCTTGGTGTAGATCAAAA, reverse primer CCACACCAGGTAGAAACTACGAAA) and human LincRNA-p21 (FAM probe ATGCGGCCTTGCAGG, forward primer CCCGGGCTTGTCTTTTGTT, reverse primer GAGTGGGTGGCTCACTCTTCTG) was performed using Custom TaqMAN Gene Expression Assays (Life Technolgies).^31^ Additional mouse TaqMAN RT-PCR assays include p53 Mm441964_g1, Gapdh Mm99999915_g1, *β*-Actin Mm00607939_s1, Noxa Mm00451763_m1, Atf2 Mm00833804_g1, Survivin Mm00599749_m1, Mcl1 Mm00725832_s1, Bax Mm00432051_m1. Puma Mm00519268_m1, Cdkn1a Mm00432448_m1 and Stat3 Mm01219775_m1. Additional human TaqMAN RT-PCR assays include GAPDH Hs33929097_g1, *β*-Actin Hs99999903_m1, NOXA Hs00560402_m1, ATF2 Hs01095345_m1, CDKN1A Hs00355782_m1 and STAT3 Hs01047580_m1 (Life Technologies). TaqMan Gene Expression Assays were used in combination with FastStart Universal Probe Master Mix (Roche). Data were analyzed using the comparative ΔΔ*C*_T_ method.

### Detection of apoptotic keratinocytes

H&E-stained mouse skin sections were used to quantify the presence of apoptotic keratinocytes. As previously described, apoptotic keratinocytes in the interfollicular basal epidermis were scored positive if all three of the following criteria were present: dark pyknotic nuclei, cytoplasmic eosinophilia and absence of cellular contacts.^51^ Data are presented as the average number of apoptotic interfollicular basal epidermal keratinocytes/cm skin.

### BrdU staining

Cells were pulsed-labeled with 10 *μ*M BrdU for 1 h before collection. Harvested cells were washed twice with ice-cold PBS and then fixed by the addition of ice-cold 75% ethanol. Staining with anti-BrdU FITC was performed as previously described.^47^ Cells were analyzed by flow cytometry at the NCSU Flow Cytometry and Cell Sorting Laboratory.

### Annexin-V staining

Harvested cells were washed twice with ice-cold PBS and then resuspended in Annexin V Binding Buffer (BioLegend, San Diego, CA, USA) at a concentration of 1 × 10^6^ cells per ml. Five microliters of Annexin V Pacific Blue was added to 100 *μ*l of the cell suspension, followed by the addition of 10 *μ*l propidium iodide (PI) solution (BioLegend). Cells were incubated for 15 min at room temperature in the dark, followed by the addition of 400 *μ*l Annexin V Binding Buffer. Cells were analyzed by flow cytometry at the NCSU Flow Cytometry and Cell Sorting Laboratory. Data were collected and presented on a scatter plot with Annexin V Pacific Blue intensity on the *x* axis and PI intensity on the *y* axis.

### Statistical analysis

Differences between groups were evaluated by two-sided *t*-tests for paired data with the significance level set to *P*<0.05.

## Abbreviations

lncRNA: long noncoding RNA
lincRNA: long intergenic noncoding RNA
NMSC: nonmelanoma skin cancer
UVB: ultraviolet B
SCC: squamous cell carcinoma
NHEK: normal human epidermal keratinocyte
K5: keratin 5
siRNA: small interfering RNA
FACS: fluorescent automated cell sorting
PI: propidium iodide
H&E: hematoxylin and eosin
FITC: fluorescein isothiocyanate
BrdU: bromodeoxyuridine
ncRNA: noncoding RNA
MEFs: mouse embryo fibroblasts
MEM: minimal erythema dose
GWAS: genome wide association studies
